# Host-associated differences in morphometric traits of parasitic larvae *Hirsutiella zachvatkini* (Actinotrichida: Trombiculidae)

**DOI:** 10.1007/s10493-015-9925-0

**Published:** 2015-05-23

**Authors:** Hanna Moniuszko, Grzegorz Zaleśny, Joanna Mąkol

**Affiliations:** Institute of Biology, Department of Invertebrate Systematics and Ecology, Wrocław University of Environmental and Life Sciences, Kożuchowska 5b, 51-631 Wrocław, Poland

**Keywords:** Chigger mites, Morphology, COI, Hosts, Rodents, Phenotypic plasticity

## Abstract

Examination of host-associated variation in the chigger mite *Hirsutiella zachvatkini* (Schluger) revealed morphological differences among larvae infesting sympatric hosts: *Apodemus agrarius*, *Apodemus flavicollis* and *Myodes glareolus*. The analysis included 61 variables of larvae obtained from their gnathosoma, idiosoma and legs (measurements and counts). Statistically significant differences were observed for metric characters of the legs as opposed to the scutum. In view of the conspecificity of the mites, supported by comparison of COI gene products obtained from larvae and laboratory-reared deutonymphs, the observed variation is attributed to phenotypic plasticity. The knowledge of larval morphology, including intraspecific variation of metric characters, supported by molecular and host range data, places *H. zachvatkini* among the most comprehensively defined members of Trombiculidae.

## Introduction

Trombiculidae sensu Goff (1999) comprise ca. 3000 species, with the vast majority (about 90 %) known exclusively from larvae. Morphology-based methods of species identification, fragmentary knowledge of phenotypic plasticity, scarcity of distributional data, and descriptions based on larvae, make it difficult to evaluate the actual number of species. The difficulties in species delimitation stem also from incomplete knowledge of host spectra and possible host-driven intra-population differences.

Despite observed morphological differences among *Psoroptes* skin mites (Astigmata: Psoroptidae), Pegler et al. (2005) found no molecular evidence of species-level diversity and thus refuted the earlier concept of distinct specific identity of the parasites from different host species. Data on host-associated differences among trombiculids are very scarce. Menezes et al. (2011) failed to find any significant morphological differences between groups of *Eutrombicula alfreddugesi* (Oudemans), which infested different species of lizards, whereas Kuo et al. (2011) observed differences in the degree of engorgement (inferred from idiosoma length and width) of *Leptotrombidium imphalum* Vercammen-Grandjean and Langston within and among its three host species.

*Hirsutiella zachvatkini*, widely distributed in Europe and Asia, is regarded as one of the most common chigger species. Its presumably wide host spectrum includes rodents, insectivores, lagomorphs and birds (Kudryashova 1998). Active postlarval forms of *H. zachvatkini* have been re-described by Daniel (1961). Data on metric and meristic characters of larvae have been provided by Stekolnikov (2001a), who has also dealt with chaetotactic anomalies and intraspecific variation of *Hirsutiella* spp. (Stekolnikov 2001b, 2003), and also by Imaz et al. (2005), however the host-induced variability was not explicitly examined.

Here we provide the results of morphometric and molecular analyses of larvae of *H. zachvatkini*, collected from striped field mouse, *Apodemus agrarius* (Pallas) (Muridae), yellow-necked mouse, *Apodemus flavicollis* (Melchior) and bank vole, *Myodes glareolus* (Schreber) (Cricetidae). Our study aims at answering the question of potential differences between mites infesting different host species.

## Materials and methods

Ectoparasitic larvae (total: 133 specimens) were collected from *A. agrarius* (46 larvae/11 host specimens), *A. flavicollis* (45/10) and *M. glareolus* (42/9). The hosts were captured in Sherman traps (permissions 66/2012, 27/2013 and 41/2013 issued by the Second Local Commission for Animal Experiments) in a deciduous forest stand in the Syców Municipal Park (51°17′22.672″N, 17°42′39.766″E), Poland, between September 2012 and April 2014. The larvae were preserved in 96 % ethanol.

A molecular analysis, aiming at evaluating the differences between the examined specimens, was carried out on three larvae and three deutonymphs (reared from the most engorged larvae). Each pair (larva + deutonymph which developed from engorged larva) originated from a different host species. Total genomic DNA was extracted using DNeasy Blood and Tissue Kit (Qiagen). The mites were transferred from 96 % ethanol to ATL lysis buffer with Proteinase K and incubated overnight at 56 °C. After digestion, the lysis buffer containing nucleic acids was transferred to a new Eppendorf tube and stored for DNA isolation according to the manufacturer’s protocol. Amplification of the DNA barcode region (cytochrome c oxidase 1 subunit) was performed using degenerate primers: bcdF04 (5′–CATTTTCHACTAAYCATAARGATATTGG–3′) and bcdR04 (5′–TATAAACYTCDGGATGNCCAAAAAA–3′) (Dabert et al. 2010) with the following thermocycling conditions: 95 °C/3 min—initial denaturation; 95 °C/30 s, 48 °C/30 s, 72 °C/45 s—40 cycles; 72 °C/7 min—final extension. The PCR reaction (25 μl) was performed using the following PCR mix: 4 μl of genomic DNA, 10 mM Tris–HCl, 50 mM KCl, 1.5 mM MgCl_2_, 200 μM of each dNTP, 150 pmol of each primer and 2 units of Taq polymerase (EurX). The amplification product was purified using QIAquick PCR purification kit (Qiagen) and sequenced on both strands (Genomed, Poland). The sequences of *H. zachvatkini* isolated from analyzed host species were identical, thus only one, obtained from deutonymph that developed from larva parasitising the bank vole, was deposited in GenBank (acc. no. KR071845).

Specimens that served for morphological studies (incl. exoskeletons that remained after DNA extraction) were mounted on microscopic slides in Hoyer’s medium. Measurements and photos were taken under a Nikon Eclipse E600 compound microscope equipped with DIC and DS-Fi1 camera, using the NIS-Elements BR software. Morphological terminology follows Goff et al. (1982). All the measurements are given in micrometres (μm). The larvae were preliminarily assigned to *H. zachvatkini* based on morphological criteria (Kudryashova 1998; Stekolnikov 2001a).

Our morphological analysis identified 61 characters of the gnathosoma, idiosoma and legs. For the list of characters and explanation of symbols see Table 1. Statistical analysis was carried out using Statistica 10 software (StatSoft 2011). Prior to the analysis the data were log-transformed (log_10_). Mean and minimum/maximum values for all variables were calculated. Out of 61 morphological characters, 23 (Ch, SB, AW, PW, AP, ASB, AM, PSB, AL, PL, S, PaTr, PaFe, PaGe, PaTi, PaTa, Odo, Leg I, Leg II, Leg III, dmt, fV, fD) were selected for discriminant function analysis (DFA). Since some of the characters listed in Table 1 were not independent, we preselected the variables and DFA was restricted to those, which were measured directly.

**Table 1:** Standard quantitative data on *Hirsutiella zachvatkini*

Character	Present study (n = 133)						After Stekolnikov (2001a)		After Imaz et al. (2005)	
	*Apodemus agrarius*		*Apodemus flavicollis*		*Myodes glareolus*					
Sample size	n = 46		n = 45		n = 42		n = 41		n = 11	
	Min.–max.	Mean	Min.–max.	Mean	Min.–max.	Mean	Min.–max.	Mean	Min.–max.	Mean
Gnathosoma										
GL	74–106	88	72–114	87	70–111	87	–	–	–	–
GW	69–119	82	68–101	82	70–110	82	–	–	–	–
Ch	35–48	42	35–49	42	33–49	43	–	–	–	–
PaTr	13–26	20	15–25	19	15–24	19	–	–	–	–
PaFe	13–28	20	13–28	20	12–26	19	–	–	–	–
PaGe	8–19	13	10–17	14	10–18	16	–	–	–	–
PaTi	8–19	11	8–19	11	8–16	10	–	–	–	–
PaTa	13–20	17	13–22	17	12–20	16	–	–	–	–
Odo	21–31	27	23–32	27	20–33	28	–	–	–	–
Idiosoma										
IL	214–769	494	259–804	535	252–787	421	–	–	–	–
IW	193–592	343	195–586	374	199–643	302	–	–	–	–
DS min.	51–60	55	51–58	54	51–57	54	41–63	50	65–75	70
DS max.	61–67	64	61–69	65	61–69	64	58–77	70	80–90	84
VS min.	26–37	30	22–32	28	24–31	27	29–53	33	33–60	39
VS max.	51–57	54	51–58	55	51–57	54	38–68	61	45–78	67
H	67–79	73	70–79	74	70–77	74	59–85	75	93–100	96
AM	42–57	50	44–57	51	41–59	51	47–60	54	50–63	57
AL	41–59	54	46–61	54	46–57	52	45–63	55	60–68	63
PL	64–84	73	66–85	74	60–84	73	67–87	74	88–106	99
AW	70–98	76	60–80	74	67–83	75	70–82	76	73–83	78
PW	78–100	87	77–91	86	76–90	86	78–95	88	93–103	96
AP	20–33	28	25–34	28	25–32	28	24–33	29	30–38	33
P-PL	19–33	25	19–31	26	18–31	24	25–34	29	–	–
S	84–123	96	90–131	99	81–111	94	86–108	96	88–100	96
SB	31–40	34	30–36	33	30–37	33	29–37	33	31–38	34
ASB	26–38	35	32–44	35	29–38	35	41–48	44	39–45	41
PSB	12–26	17	14–21	17	13–26	17	14–19	17	15–18	16
SD	40–57	51	48–63	52	46–59	51	57–66	61	54–60	58
fD	86–96	90	82–96	88	88–96	91	73–98	86	68–95	76
fV	52–58	55	52–72	58	52–64	57	56–91	73	64–112	92
NDV	138–154	146	136–166	146	142–158	148	145–180	160	150–185	168
Legs										
Cx I	68–90	76	72–91	79	71–86	79	–	–	–	–
Tr I	25–42	33	30–43	35	25–40	29	–	–	–	–
bFe I	33–45	39	31–46	40	31–42	37	–	–	–	–
tFe I	27–43	35	30–44	36	29–39	34	–	–	–	–
Ge I	32–46	38	30–46	39	34–46	39	–	–	–	–
Ti I	39–49	44	38–49	44	40–51	44	–	–	–	–
Ta I L	65–87	78	69–86	78	68–85	79	–	–	–	–
Ta I W	21–34	26	22–37	26	22–31	26	–	–	–	–
Leg I	309–448	359	317–460	370	70–98	77	326–382	359	337–386	363
Cx II	68–87	77	72–95	80	70–98	77	–	–	–	–
Tr II	25–40	32	25–45	33	22–40	30	–	–	–	–
bFe II	24–30	33	29–42	35	23–38	31	–	–	–	–
tFe II	22–33	28	20–35	28	20–31	26	–	–	–	–
Ge II	21–35	29	24–36	28	21–39	28	–	–	–	–
Ti II	35–45	40	36–47	41	35–49	41	–	–	–	–
Ta II L	61–79	71	64–80	72	63–79	71	–	–	–	–
Ta II W	19–26	23	19–28	23	20–28	24	–	–	–	–
Leg II	284–409	323	294–408	333	326–401	360	301–355	331	327–356	342
Cx III	54–78	67	60–81	70	60–79	67	–	–	–	–
Tr III	28–46	37	30–50	39	27–50	35	–	–	–	–
bFe III	32–46	40	31–46	39	30–49	36	–	–	–	–
tFe III	23–35	29	23–36	29	25–35	39	–	–	–	–
Ge III	22–36	30	24–35	30	22–38	31	–	–	–	–
Ti III	38–58	50	45–56	50	36–50	51	–	–	–	–
Ta III L	80–97	90	83–101	91	82–99	90	86–103	95	101–115	110
Ta III W	14–23	19	17–25	20	16–22	19	18–22	19	20–23	21
Leg III	316–438	358	320–448	365	378–470	404	342–391	371	356–396	382
IP	914–1293	1040	959–1305	1068	1102–1325	1179	997–1120	1061	1020–1119	1087
dmt	19–30	25	18–28	26	18–28	23	–	–	–	–
mt	0.213–0.336	0.277	0.212–0.313	0.260	0.191–0.306	0.253	0.221–0.314	0.271	0.244–0.289	0.267

List of abbreviations (symbols apply to length, unless stated otherwise): GL—gnathosoma, GW—width of gnathosoma, Ch—cheliceral blade, PaTr—palpal trochanter, PaFe—palpal femur, PaGe—palpal genu, PaTi—palpal tibia, PaTa—tarsus, Odo—palp tibial claw (odontus); idiosoma (excl. scutum): IL—idiosoma, IW—width of idiosoma, DS—dorsal idiosomal setae, VS—ventral idiosomal setae, H—humeral seta, fD—number of dorsal idiosomal setae (excl. setae on scutum), fV—number of ventral idiosomal setae; scutum: AM—antero-medial seta, AL—anterolateral seta, PL—postero-lateral seta, AW—distance between bases of AL setae, PW—distance between bases of PL setae, AP—distance between bases of AL and PL (on one side of symmetry axis), P-PL—distance between postero-lateral seta (PL) and posterior margin of scutum, S—sensilla, SB—distance between bases of sensillae, ASB—distance between the level of sensillae (S) and anterior margin of scutum, PSB—distance between the level of sensillae (S) and posterior margin of scutum, SD—scutum (=ASB + PSB); legs: Cx—coxa, Tr—trochanter, bFe—basifemur, tFe—telofemur, Ge—genu, Ti—tibia, Ta (…) L—tarsus (including pretarsus), Ta (…) W—width of tarsus, Leg (…)—total length of leg, IP (*index pedibus*, leg index)—total length of legs on one side of symmetry axis, dmt—distance between the base of mastitarsala and proximal margin of tarsus III, mt—dmt/Ta III L

## Results

Sequencing of the COI gene yielded six identical barcode sequences of 680 bp. We did not observe any nucleotide substitutions in this region, and no intraspecific variation at molecular level could be confirmed.

The ranges of larval characters used in the present study and those examined by Stekolnikov (2001a) and Imaz et al. (2005) overlapped (Table 1), except for DS min., DS max., H and PL provided by Imaz et al. (2005), hence, in the lack of other differentiating characters, the affiliation of our material with *T. zachvatkini* could be confirmed.

**Fig. 1:**
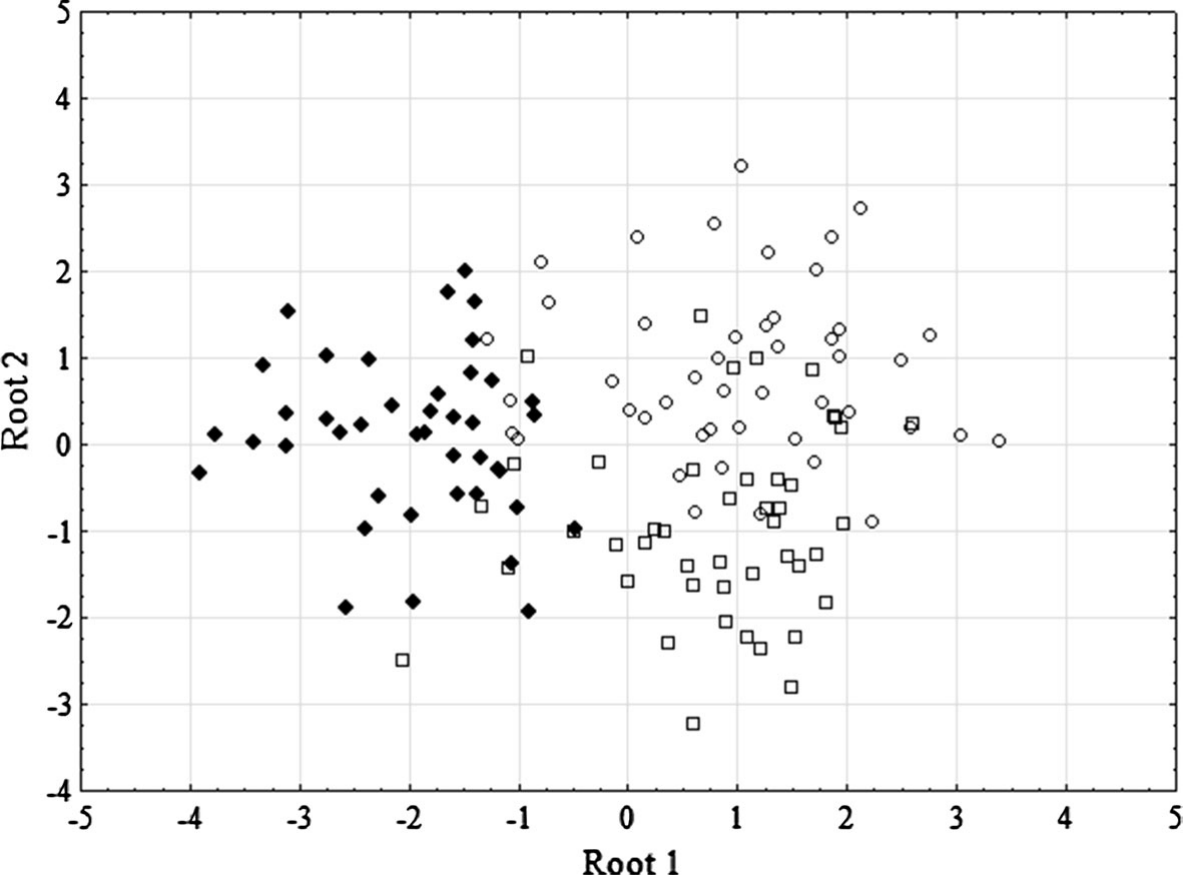
Results of canonical analysis of *Hirsutiella zachvatkini* obtained from three host species. Plot generated based on 23 variables measured in 133 specimens. *Symbols* denoting host species: *squares*—*Apodemus flavicollis*, *circles*—*Apodemus agrarius*, *black diamonds*—*Myodes glareolus*

The model generated by DFA is provided in Table 2. The Roots 1 and 2 account for 76.8 and 100 % of the total variation within *H. zachvatkini* collected from three host species. The variables that play the major role in this differentiation are, in descending order, Leg III, PaTa, PaTi, S, dmt and PSB. The means of canonical values (Table 3) indicate that Root 1 discriminates the specimens of *H. zachvatkini* obtained from *M. glareolus.* When the canonical scores from the discriminant analysis are plotted and viewed (Fig. 1), it can be seen that representatives of *H. zachvatkini* collected from *M. glareolus* [with the total percentage of correctly classified specimens accounting for 95.2 % (Table 3)] are clearly separated from the mites collected from *Apodemus* mice.

**Table 2:** Summary of discriminant function analysis across the complete list of variables

Variable	Wilks’ lambda	Partial lambda	*p* value	Root 1	Root 2
Ch	0.237	0.962	0.12	−0.269	−0.039
SB	0.235	0.973	0.23	0.113	0.359
AW	0.240	0.952	0.070	−0.151	0.490
PW	0.231	0.986	0.47	0.091	−0.263
AP	0.228	0.999	0.96	−0.012	−0.055
ASB	0.233	0.980	0.33	−0.055	−0.286
AM	0.229	0.996	0.79	0.043	−0.118
PSB	0.242	0.944	0.045	0.103	−0.451
AL	0.239	0.956	0.090	0.298	−0.118
PL	0.231	0.989	0.56	−0.045	−0.193
S	0.248	0.920	0.011	0.341	−0.271
PaTr	0.229	0.997	0.85	−0.042	0.093
PaFe	0.237	0.963	0.13	0.250	0.149
PaGe	0.232	0.982	0.39	0.014	−0.245
PaTi	0.258	0.884	0.001	0.478	0.268
PaTa	0.266	0.859	<0.001	0.479	−0.290
Odo	0.237	0.965	0.14	−0.261	0.107
Leg I	0.241	0.949	0.059	−0.641	−0.051
Leg II	0.234	0.976	0.26	0.252	−0.610
Leg III	0.243	0.941	0.038	−0.731	0.475
dmt	0.243	0.938	0.032	0.272	0.327
fV	0.233	0.978	0.31	0.063	−0.261
fD	0.238	0.958	0.096	0.033	0.439
Eigenvalue				1.824	0.551
Cumulative proportion				0.768	1.000
Roots removed	Eigenvalue	Wilks’ lambda	Chi square	df	*p* value
0	1.825	0.228	175.779	46	<0.001
1	0.551	0.645	52.215	22	<0.001

Chi square tests with successive roots removed are provided in the lower part of the table. Root 1 and Root 2 columns refer to standardized coefficients of canonical variables

**Table 3:** Classification efficiency of *Hirsutiella zachvatkini* from each host species

	% of correct classification	*A. agrarius* (*p* = 0.35)	*A. flavicollis* (*p* = 0.34)	*M. glareolus* (*p* = 0.32)	Root 1	Root 2
*A. agrarius*	78.3	36	6	4	1.019	0.839
*A. flavicollis*	71.1	8	32	5	0.788	−0.930
*M. glareolus*	95.2	0	2	40	−1.961	0.077
Overall	81.2	44	40	49		

Root 1 and Root 2 columns refer to the means of canonical values

## Discussion

Identical DNA sequences obtained from the chigger specimens parasitising different host species suggest their conspecificity. This is compatible with Shatrov and Kudryashova’s (2008) view that host selection in trombiculid mites is imposed by the habitat of the larvae, which infest all available vertebrates. According to Stekolnikov and Klimov (2010) size variation may reflect differences in environmental conditions, and is not necessarily genetically-based, as opposed to qualitative traits. As stated by Traub and Wisseman (1974) trombiculid larvae during their search for host are exposed to desiccation, therefore the risk of failure in finding the suitable host makes them less host-selective, even in view of lower energetic returns (Kuo et al. 2011).

The identical COI sequences may also reflect a relatively short co-evolution of parasites and their hosts. The assumption is especially relevant in the case of parasitengone mites, which may have switched their host groups several times; for example, between insects or from insects to arachnids and, in the case of Trombiculidae, to vertebrates (Audy 1960). The wide host range and the distribution of hosts on the phylogenetic tree of parasitengone mites do not allow an exact determination of the primary host or host range in the stem lineage of the cohort (Wohltmann 2000). No doubt, vertebrate hosts offered new evolutionary possibilities and reduced the selection pressure through their morphological constitution, body mass and continuous abundance throughout the year (Wohltmann 2000). The strategy, which may reflect similar evolutionary trends, has been already recognised in other, non-chigger, mites. Baulechner’s et al. (2013) morphological and molecular (COI) analysis of host specificity in three species of *Spinturnix* (Mesostigmata: Spinturnicidae)—parasites of four sympatric bat species (*Myotis* spp.) revealed the occurrence of three major, morphologically different clades. Yet, there was no evidence for co-speciation, but host switch and sorting event were confirmed. Furthermore, the hosts were several million years older than their parasites.

The morphological differences among the chiggers collected from the bank vole and mice should be regarded as intraspecific variation, which is induced by the host and thus reflects the adaptation to local microenvironment. Pegler et al. (2005) studied putative species of *Psoroptes* (Psoroptidae), associated with different host taxa. Their morphological and molecular (ITS-2 gene sequence) analyses showed that the observed variation was insufficient to consider the mites as representing distinct species. Nevertheless, the conclusions of Pegler et al. (2005) and those resulting from our study are not supported by the same strategies involved in host–parasite associations. In the case of psoroptids, the whole life cycle occurs on the host, whereas in Trombiculidae the contact with the host is limited to the larva only. The different selection pressures on the larval instar may be important for further conclusions. Contrary to most other parasitengones, the duration of contact with the host in trombiculids may go beyond the actual phase of parasitism. Traub et al. (1975), observed larvae of *Leptotrombidium* spp. associated with their hosts (op. cit. “chiggers would wander for hours, or a day or longer”) before the onset of feeding. The latter, besides the knowledge of local adaptation of larvae to occupy particular places within the host body, may contribute to finding the background for the most pronounced morphological differences in the length of leg segments among the chiggers collected from the bank vole and mice. It cannot be excluded that during the prolonged contact with host, the neosomy, i.e. additional production of cuticle without intermittent moult, the phenomenon described by Audy et al. (1972) for *Vatacarus* (Trombiculidae) and reported also by Wohltmann (1999) in relation to non-trombiculid parasitengone mites, may occur. As opposed to legs, we observed a relatively small and statistically insignificant variation of the morphological characters of the scutum. The lack of differences in scutal traits is compatible with the results obtained by Menezes et al. (2011) for *Eutrombicula alfreddugesi* collected from various lizard species. The authors compared six metric characters of the scutum in *E. alfreddugesi* collected from four species of *Tropidurus* spp. (Reptilia: Squamata).

Despite the fact that further studies should focus on retracing the ecological background and consequences of host–parasite association, the present knowledge of variation of metric characters in larvae of *H. zachvatkini* (Table 1), supported by qualitative and meristic characters, molecular data and host range data, allows to place the species in question among the most comprehensively defined members of Trombiculidae.

## References

[CR1] Audy JR (1960) Evolutionary aspects of trombiculid mite parasitism. In: Purchon RD (ed.) Proceedings of the century and bicentury Congress of Biology. University of Malaya press, Singapore, pp 102–108

[CR2] Audy JR, Radovsky FJ, Vercammen·Grandjean PH (1972). Neosomy: radical intrastadial metamorphosis associated with arthropod symbiosis. J Med Entomol.

[CR3] Baulechner D, Becker NI, Encarnaçāo J (2013). Host specificity in spinturnicid mites: do parasites share a long evolutionary history with their host?. J Zool Syst Evol Res.

[CR4] Dabert M, Witaliński W, Kaźmierski A, Olszanowski Z, Dabert J (2010). Molecular phylogeny of acariform mites (Acari, Arachnida): strong conflict between phylogenetic signal and long-branch attraction artifacts. Mol Phylogenet Evol.

[CR5] Daniel M (1961). Contribution a la connaissance des formes adultes des Trombiculidae d’Europe. I. Description des nymphes et des adultes du *Trombicula* (*N*.) *zachvatkini* Schluger 1948 et *Trombicula* (*N*.) *talmiensis* Schluger 1955. Acarologia.

[CR6] Goff ML, Needham GR, Mitchell R, Horn DJ, Welbourn WC (1999). The current state of chigger systematics: a view from a swamp 20 km SSE of Eden. Acarology IX.

[CR7] Goff ML, Loomis RB, Welbourn WC, Wrenn WJ (1982). A glossary of chigger terminology (Acari: Trombiculidae). J Med Entomol.

[CR8] Imaz A, Galicia D, Moraza ML, Stekolnikov AA (2005). Contribution to the knowledge of chigger mites (Acari: Trombiculidae) parasitizing *Apodemus sylvaticus* (L.) (Rodentia, Muridae) on the Iberian Peninsula. Acarologia.

[CR9] Kudryashova NI (1998). Chigger mites (Acariformes, Trombiculidae) of East Palearctics.

[CR10] Kuo C-C, Wang H-C, Huang C-L (2011). Variation within and among host species in engorgement of larval trombiculid mites. Parasitology.

[CR11] Menezes VA, Fontes AF, Gettinger D, Van Sluys M, Rocha CFD (2011). A morphometric study of *Eutrombicula alfreddugesi* (Acari: Trombiculidae) infesting four sympatric species of *Tropidurus* (Squamata: Tropiduridae) in northeastern Brazil. Phyllomedusa.

[CR12] Pegler KR, Evans L, Stevens JR, Wall R (2005). Morphological and molecular comparison of host derived populations of parasitic Psoroptes mites. Med Vet Entomol.

[CR13] Shatrov AB, Kudryashova NI (2008). Taxonomic ranking of major trombiculid subtaxa with remarks on the evolution of host–parasite relationships (Acariformes: Parasitengona: Trombiculidae). Ann Zool.

[CR14] StatSoft (2011) STATISTICA (data analysis software system), version 10. www.statsoft.com

[CR15] Stekolnikov AA (2001). Systematics of chigger mites of the genus *Hirsutiella* Schluger et Vysotzkaya, 1970 (Acari: Trombiculidae). Entomol Rev.

[CR16] Stekolnikov AA (2001). Intraspecific variance of chaetotactic characters in the chigger mite genus *Hirsutiella* (Acari: Trombiculidae). Parazitologiya.

[CR17] Stekolnikov AA (2003). Intraspecific variability and sympatria in closely related chigger mites species of the genus *Hirsutiella* (Acari: Trombiculidae). Parazitologiya.

[CR18] Stekolnikov AA, Klimov P (2010). A revision of chiggers of the minuta species-group (Acari: Trombiculidae: *Neotrombicula* Hirst, 1925) using multivariate morphometrics. Syst Parasitol.

[CR19] Traub R, Wisseman CL (1974). The ecology of chigger-borne rickettsiosis (scrub typhus). J Med Entomol.

[CR20] Traub R, Wisseman CL, Jones MR, O’Keefe JJ (1975). The acquisition of *Rickettsia tsutsugamushi* by chiggers (trombiculid mites) during the feeding process. Ann N Y Acad Sci.

[CR21] Wohltmann A, Bruin J, van der Geest LPS, Sabelis M (1999). Life history evolution in Parasitengonae (Acari: Prostigmata): constraints on number and size of offspring. Evolution and ecology of Acari.

[CR22] Wohltmann A (2000). The evolution of life histories in Parasitengona (Acari: Prostigmata). Acarologia.

